# Transcriptome analysis identifies genes involved in the somatic embryogenesis of Eucalyptus

**DOI:** 10.1186/s12864-020-07214-5

**Published:** 2020-11-18

**Authors:** Yufei Xiao, Junji Li, Ye Zhang, Xiaoning Zhang, Hailong Liu, Zihai Qin, Bowen Chen

**Affiliations:** Guangxi Key Laboratory of Superior Timber Trees Resource Cultivation, Guangxi Forestry Research Institute, 23 Yongwu Road, Nanning, 530002 Guangxi China

**Keywords:** Eucalyptus, Vegetative propagation, Somatic embryogenesis, Dedifferentiation, Transcriptome, Callus

## Abstract

**Background:**

Eucalyptus, a highly diverse genus of the *Myrtaceae* family, is the most widely planted hardwood in the world due to its increasing importance for fiber and energy. Somatic embryogenesis (SE) is one large-scale method to provide commercial use of the vegetative propagation of Eucalyptus and dedifferentiation is a key step for plant cells to become meristematic. However, little is known about the molecular changes during the Eucalyptus SE.

**Results:**

We compared the transcriptome profiles of the differentiated and dedifferentiated tissues of two Eucalyptus species – *E. camaldulensis* (high embryogenetic potential) and *E. grandis x urophylla* (low embryogenetic potential). Initially, we identified 18,777 to 20,240 genes in all samples. Compared to the differentiated tissues, we identified 9229 and 8989 differentially expressed genes (DEGs) in the dedifferentiated tissues of *E. camaldulensis* and *E. grandis x urophylla*, respectively, and 2687 up-regulated and 2581 down-regulated genes shared. Next, we identified 2003 up-regulated and 1958 down-regulated genes only in *E. camaldulensis*, including 6 somatic embryogenesis receptor kinase, 17 ethylene, 12 auxin, 83 ribosomal protein, 28 zinc finger protein, 10 heat shock protein, 9 histone, 122 cell wall related and 98 transcription factor genes. Genes from other families like ABA, arabinogalactan protein and late embryogenesis abundant protein were also found to be specifically dysregulated in the dedifferentiation process of *E. camaldulensis*. Further, we identified 48,447 variants (SNPs and small indels) specific to *E. camaldulensis*, including 13,434 exonic variants from 4723 genes (e.g., annexin, GN, ARF and AP2-like ethylene-responsive transcription factor). qRT-PCR was used to confirm the gene expression patterns in both *E. camaldulensis* and *E. grandis x urophylla*.

**Conclusions:**

This is the first time to study the somatic embryogenesis of Eucalyptus using transcriptome sequencing. It will improve our understanding of the molecular mechanisms of somatic embryogenesis and dedifferentiation in Eucalyptus. Our results provide a valuable resource for future studies in the field of Eucalyptus and will benefit the Eucalyptus breeding program.

**Supplementary Information:**

The online version contains supplementary material available at 10.1186/s12864-020-07214-5.

## Background

Eucalyptus is a highly diverse genus of the *Myrtaceae* family and is widely planted across the world for its increasing importance for timber and pulp [1]. Natural regeneration of Eucalyptus mainly relies on seeds, however, their breeding is always a slow process due to the length of the juvenile phase [1]. Thus, vegetative propagation becomes an alternative option for Eucalyptus to breed and to gain new characteristics. Vegetative propagation consists of methods of both micropropagation (e.g., air layering, grafting, rooting of cuttings) and micropropagation using in vitro tissue culture techniques, including adventitious budding, axillary shoot tips, somatic embryogenesis (SE) [2]. Among them, in vitro tissue culture-induced SE, in which whole fertile plants are regenerated under proper culture conditions, is widely used to propagate selected genotypes for commercial purposes because it can provide large-scale regenerations for plants with relative lower cost [2].

In forest trees, SE is a complex process involving many factors in different steps [2]. The first step is the callus induction in which differentiated somatic cells (e.g., seed, leaf, stem) acquire embryogenetic competence with or without a dedifferentiation step [3]. In Eucalyptus, Ouyang et al. reported the SE from the callus of seedlings for the first time in 1980 [4]. Pinto reviewed the status and future perspectives of SE in Eucalyptus [5]. Some tissues (e.g., hypocotyls, cotyledons, leaves, shoots) from both young seedlings and old trees have been reported for successful induction of callus in multiple Eucalyptus species, such as *E. botryoides*, *E. dunnii*, *E. grandis*, *E. globulus* and *E. rudis* [5]. Including the culture medium, some other factors have been reported to affect the SE induction, such as the addition of antioxidants, the carbon source, the effect of genotype and the morphogenic pathway [5]. It is clear that the capacity of SE in Eucalyptus species varies, however, our knowledge about the genomic and transcriptomic information controlling the SE and vegetative propagation is still poor.

Some studies have reported genetics associated with the capacity of vegetative propagation in Eucalyptus. For example, Grattapaglia used a pseudo-testcross strategy and RAPD markers to study the quantitative trait loci (QTLs) controlling vegetative propagation in *E. grandis* and *E. urophylla* [6]. Marques identified some QTLs related to adventitious rooting, sprouting ability and the stability of adventitious rooting [7]. Thumma identified some QTLs related to the wood quality in *Eucalyptus nitens* [8]. Ohtani summarized the impacts of the regulation of RNA metabolism for in vitro dedifferentiation of plant cells, such as rRNA biosynthesis, pre-mRNA splicing, and miRNA-based RNA decay [9]. In addition, gene regulation in SE has been studied in plants, such as Arabidopsis [10], alfalfa [11], camphor tree [12], carrot [13], cotton [14–16], orange [17], potato [16] and soybean [18–20]. Many genes have been reported to function in SE, such as auxin response factor19 (ARF19), somatic embryogenesis receptor-like kinase (SERK), leafy cotyledon (LEC), baby boom (BBM), and wuschel (WUS) [21, 22]. However, it is still unknown about the gene changes and gene regulations in the SE and dedifferentiation of Eucalyptus.

In the present study, we analyzed the transcriptome profiles and gene variants in the dedifferentiation process of two Eucalyptus species – *E. camaldulensis* (high embryogenetic potential) and *E. grandis x urophylla* (low embryogenetic potential). Some genes were identified to relate to the SE and dedifferentiation of Eucalyptus, such as auxin related genes, embryogenesis related genes, ethylene related genes, ribosomal protein (RP) genes, zinc finger protein (ZFP) genes, histone genes, heat shock protein (HSP) genes and various transcription factors (TFs). Further, we identified functional variants in these two Eucalyptus species that might control the callus induction and development. This is the first time to report the SE transcriptomes in Eucalyptus. It will improve our understanding about the molecular mechanisms in the SE of Eucalyptus. Further, the output of this study will provide a valuable resource for future studies and will benefit the research in this field, especially the breeding program of Eucalyptus.

## Results

### Morphological characterization of somatic callus

**Fig. 1:**
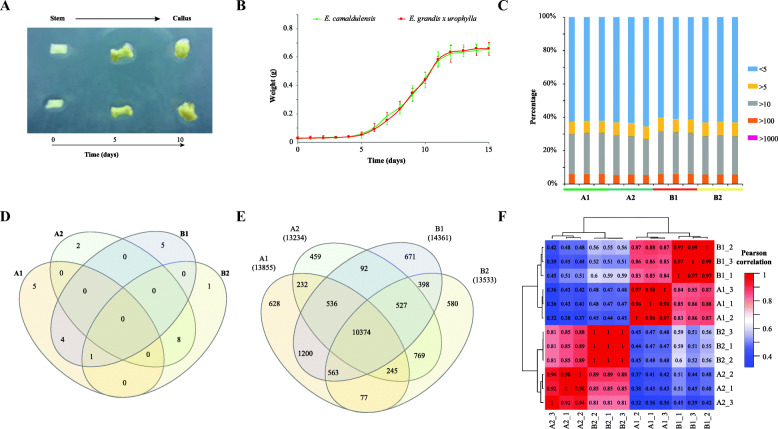
Morphological characterization of somatic callus and overview of the transcriptome sequencing. **a** Morphological characterization of the dedifferentiation from stem to callus. Upper panel: *E. camaldulensis*; lower panel: *E. grandis x urophylla*. **b** Growth curves of the callus tissue of *E. camaldulensis* and *E. grandis x urophylla*. **c** Number of genes identified in the stem and callus of *E. camaldulensis* (A1, A2) and *E. grandis x urophylla* (B1, B2). **d** Venn diagram of top 10 highly expressed genes in all samples. **e** Venn diagram of genes (TPM > 1) identified in all samples. **f** Heat map of sample correlation based on the gene expression profiles. Color bar represents the values of Pearson correlation

To study the somatic embryogenesis of Eucalyptus, the stem (differentiated) segments of both *E. camaldulensis* (A1) and *E. grandis x urophylla* (A2) were tissue-culture induced to callus (B1 for *E. camaldulensis* and B2 for *E. grandis x urophylla*). In these two Eucalyptus species, the morphologies (Fig. 1a) of the tissue culture induced callus were similar and their growth curves (Fig. 1b) showed S-shaped during dedifferentiation. The weight and appearance of the stem had no significant change during the first 2 days of induction. After 3 to 6 days induction, the two ends of the stem started to be induced to callus, which showed that both ends turned pale yellow, expanded and thickened like dumbbell (Fig. 1a). Then, it fell into the rapid growth period of 7 to 12 days induction. The dedifferentiated callus gradually extended from both ends to the middle and the whole stem was completely dedifferentiated into pale yellow and moist callus. After 13 days of induction, it entered into the slow growth stage. The callus which had been cultured in the induction medium for 10 days were used for transcriptome sequencing.

### Transcriptome sequencing and gene expression profiles

Next, we analyzed the transcriptome profiles of differentiated and dedifferentiated tissues of *E. camaldulensis* and *E. grandis*. Table 1 showed an overview of the transcriptome sequencing and the numbers of genes identified in each sample. Initially, we obtained 528 million reads for all 12 samples (in triplicates, *n* = 3) with an average of 44 million reads. Then, the reads were aligned to the *E. grandis* genome and it showed that 64.66 to 74.84% of the total reads were mapped. Out of the total 36,349 *E. grandis* genes, we identified 18,777 to 20,240 genes with TPM > 1 in the differentiated and dedifferentiated tissues of Eucalyptus (Additional file 1). When we filtered genes using TPM < 5, only 12,650 to 14,464 genes were identified. Figure 1c showed that more than 60% of the genes were lowly expressed (TPM < 5) and only 0.165 to 0.272% of the Eucalyptus genes were expressed higher than 1000 TPM. Interestingly, highly expressed genes varied in differentiated and dedifferentiated tissues of Eucalyptus. Figure 1d showed the comparison of top 10 highly expressed genes in all samples according to the average TPM. Dedifferentiated tissues of both *E. camaldulensis* and *E. grandis x urophylla* shared 8 highly expressed genes including Eucgr.H01085 (ethylene-responsive transcription factor ERF071), Eucgr.G03106 (wound-induced protein 1) and Eucgr.F00114 (zinc finger protein ZAT10). We next compared the genes identified in all the samples (TPM > 1) and found 10,374 genes shared (Fig. 1e). In addition, 628, 459, 671 and 580 genes were specifically identified in A1, A2, B1 and B2, respectively. Based on the gene expression we analyzed the correlation between samples. It was revealed that replicates were performed well and that differentiated tissues were distinguishable from dedifferentiated tissues in *E. camaldulensis* and *E. grandis x urophylla* (Fig. 1f).

**Table 1:** Overview of transcriptome sequencing and gene expression profiles

Sample name	Total reads	Mapped reads	Percentage	Genes (TPM > 1)	Genes (TPM > 5)
A1_1	43,673,352	32,135,161	73.58%	18,777	13,561
A1_2	43,658,680	32,673,328	74.84%	19,024	13,855
A1_3	43,743,786	30,350,588	69.38%	19,115	13,858
A2_1	41,999,356	27,550,545	65.60%	18,950	13,435
A2_2	43,718,576	28,266,987	64.66%	19,094	13,360
A2_3	41,978,434	27,287,763	65.00%	18,406	12,650
B1_1	45,524,984	32,704,591	71.84%	20,240	14,464
B1_2	43,753,980	31,740,739	72.54%	20,186	14,274
B1_3	45,492,018	32,752,661	72.00%	19,942	14,147
B2_1	43,661,620	32,556,137	74.56%	19,322	13,518
B2_2	47,151,700	35,051,250	74.34%	19,338	13,567
B2_3	43,651,908	32,069,801	73.47%	19,276	13,536

### DEGs in *E. camaldulensis*

**Fig. 2:**
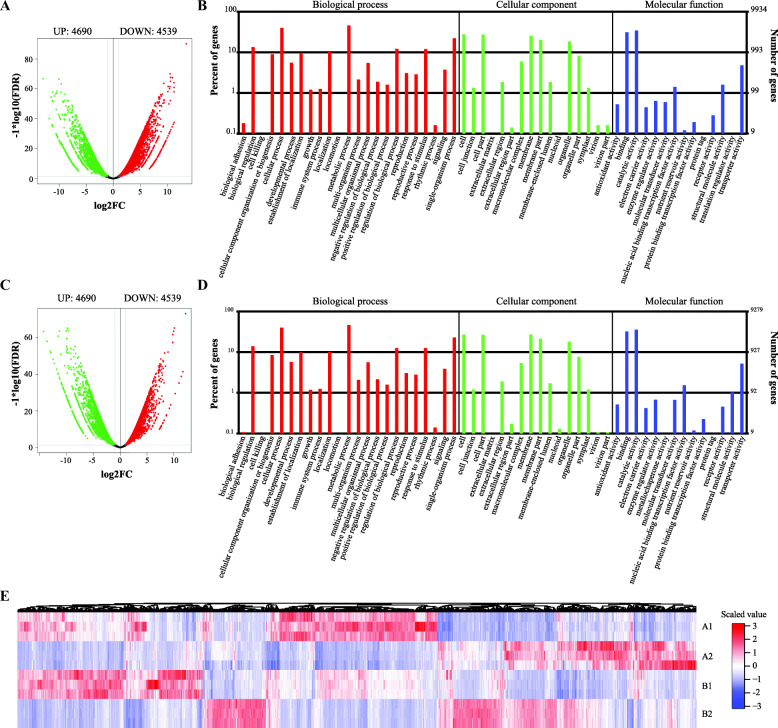
DEGs identified in the callus compared to the stem of *E. camaldulensis* and *E. grandis x urophylla*. **a** Volcano plot showing up- and down-regulated genes in the callus of *E. camaldulensis* compared to the stem. **b** Gene Ontology analysis for the DEGs identified in *E. camaldulensis*. **c** Volcano plot of the DEGs identified in the callus of *E. grandis x urophylla* compared to the stem. **d** Gene Ontology analysis of DEGs identified in *E. grandis x urophylla*. € Heat map of all DEGs identified in *E. camaldulensis* and *E. grandis x urophylla* showing the genes expression patterns in the dedifferentiation process

To study the possible functional genes/pathways in the SE of Eucalyptus, we first identified DEGs in the dedifferentiated tissue of *E. camaldulensis* compared to the differentiated tissue. Using edgeR we identified 4690 up-regulated and 4539 down-regulated genes (Fig. 2a, Additional file 2). It showed that Eucgr.H02264 (probable indole-3-acetic acid-amido synthetase GH3.1), Eucgr.D02625 (phosphoribulokinase, chloroplastic), Eucgr.J03055 (hypothetical protein), Eucgr.H02600 (protein SRG1) and Eucgr.B03016 (LOB domain-containing protein 40) were the top 5 up-regulated genes while Eucgr.J00794 (DNA-damage-repair/toleration protein DRT100), Eucgr.I02271 (endochitinase), Eucgr.A01080 (glycine-rich RNA-binding protein), Eucgr.D00703 (beta-galactosidase 8) and Eucgr.A01881 (trans-resveratrol di-O-methyltransferase) were the top 5 down-regulated genes, according to the FDR (false discovery rate) values (Additional file 2). We next analyzed the KEGG pathways enriched by the DEGs and found that “plant hormone signal transduction” (ko0475), “RNA transport” (ko03013) and “carbon metabolism” (ko01200) were the top 3 significant pathways. GO enrichment analysis (Fig. 2b) showed that together with the GO terms enriched by more than 10% of the DEGs, “reproduction” and “reproduction process” were found with interest.

Then, we analyzed some gene groups might be involved in the dedifferentiation process of *E. camaldulensis*. Initially, 27 up-regulated and 22 down-regulated genes were found to associate with ethylene (Table 2), including 7 AP2-like ethylene-responsive TFs, 41 ethylene-responsive TFs and 1 ethylene response sensor (Additional file 2). In addition, 41 DEGs encoding auxin related products were found in the callus of *E. camaldulensis* compared to the stem (Table 2), including 7 auxin response factors, 3 auxin-binding proteins, 2 auxin-induced in root clusters proteins and 16 auxin-responsive proteins (Additional file 2). Furthermore, we found 111, 79, 36, 39 and 272 DEGs (Table 2) encoding RP, ZFP, HSP, histone and TF, respectively, might be involved in the dedifferentiation process of *E. camaldulensis*. Notably, 8 up-regulated and 3 down-regulated embryogenesis related genes were identified in the callus of *E. camaldulensis* compared to the stem, such as late embryogenesis abundant protein, somatic embryogenesis receptor kinas 1 and embryogenesis-associated protein EMB8 (Additional file 2). Further, based on the GO annotation we identified 274 DEGs (154 up-regulated and 120 down-regulated) related to cell wall (Table 2). Details of these cell wall related DEGs in different categories, such as “GO:0009505~plant-type cell wall” and “GO:0009834~plant-type secondary cell wall biogenesis”, can be accessed in the additional file 3. Among them, 9 DEGs encoding expansin were up-regulated and 1 gene encoding PME53 (probable pectinesterase 53) were down-regulated.

**Table 2:** Genes differentially expressed in dedifferentiated tissues compared to differentiated tissues in Eucalyptus

Gene family	*E. camaldulensis*	*E. grandis x urophylla*	Shared	*E. camaldulensis* only
Ethylene related	27/22^a^	16/37	15/17	12/5
Auxin	25/16	18/18	15/14	10/2
Ribosomal protein	15/86	41/8	12/6	3/80
Zinc finger protein	35/44	38/38	28/23	7/21
Heat shock protein	23/13	29/6	21/5	2/8
Histone	27/12	33/11	24/6	3/6
TF	155/117	128/143	95/79	60/38
ABA	3/5	5/4	3/2	0/3
AGP	5/3	4/6	2/3	3/0
Embryogenesis	8/3	5/3	3/2	5/1
Cell wall	154/120	131/120	86/66	68/54

^a^Molecule and denominator represent the numbers of up- and down-regulated genes, respectively

### DEGs in *E. grandis* x urophylla

We next identified 4200 up-regulated and 4708 down-regulated genes in the dedifferentiated tissue of *E. grandis x urophylla* compared to the differentiated tissue (Fig. 2c). According to the FDR, top 5 DEGs include Eucgr.B03016 (LOB domain-containing protein 40), Eucgr.H02264 (probable indole-3-acetic acid-amido synthetase GH3.1), Eucgr.I02271 (endochitinase), Eucgr.L02894 and Eucgr.L02534 (Additional file 2). Notably, pathway analysis identified “Plant hormone signal transduction” (ko04075) significantly enriched by 260 DEGs (q-value: 8.36E-14), which indicates that these DEGs may play key roles in the SE of *E. grandis x urophylla*. GO analysis (Fig. 2d) showed that most of the GO terms involved by the DEGs were shared by *E. grandis x urophylla* and *E. camaldulensis* but some might be specific to *E. grandis x urophylla*, such as biological adhesion, virion and nucleoid.

Table 2 showed the numbers of DEGs from the seven gene groups identified in the dedifferentiation process of *E. grandis x urophylla*. Interestingly, we found more DEGs (41 up-regulated and 8 down-regulated) encoding ribosomal proteins in *E. grandis x urophylla* than *E. camaldulensis*, especially some RP genes which were up-regulated in *E. grandis x urophylla* but down-regulated in *E. camaldulensis*. In addition, compared to *E. camaldulensis* more down-regulated ethylene related genes and more up-regulated HSP and histone genes were found in *E. grandis x urophylla* (Table 2). Figure 2e showed an overview of all the DEGs identified in the dedifferentiated tissues compared to the differentiated tissues in both *E. camaldulensis* and *E. grandis x urophylla*. It showed that some DEGs were specifically identified in *E. camaldulensis* or *E. grandis x urophylla*, which might relate to the regenerative ability of Eucalyptus.

### Regenerative ability associated genes

**Fig. 3:**
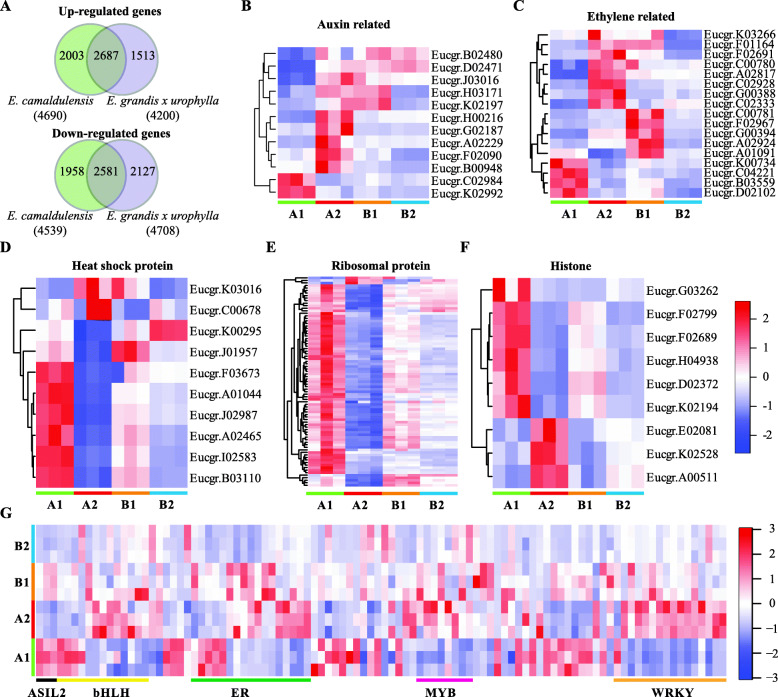
DEGs related to the SE of Eucalyptus. **a** Comparison of up-regulated (upper panel) and down-regulated (lower panel) genes identified in *E. camaldulensis* and *E. grandis x urophylla*. Heat maps of *E. camaldulensis* specific DEGs related to auxin (**b**), ethylene (**c**), heat shock protein (**d**), ribosomal prote€(**e**), histone (**f**) and transcription factor (**g**)

We next compared the DEGs identified in the dedifferentiation process of *E. camaldulensis* and *E. grandis x urophylla*. It showed in the upper panel of Fig. 3a that they shared 2687 up-regulated genes in the dedifferentiated tissue compared to differentiated tissue, including Eucgr.H01085 (ethylene-responsive transcription factor ERF071), Eucgr.A01538 (fructose-bisphosphate aldolase 6, cytosolic), Eucgr.G03106 (wound-induced protein 1), Eucgr.K02614 (NDR1/HIN1-Like protein 3), Eucgr.F00114 (zinc finger protein ZAT10) and Eucgr.H03082 (early nodulin-75) (Additional file 2). There were 2003 and 1513 up-regulated genes specifically identified in the callus of *E. camaldulensis* and *E. grandis x urophylla*, respectively (upper panel of Fig. 3a). Then, we compared to down-regulated genes and found that they shared 2581 genes (lower panel of Fig. 3a), including Eucgr.J00025 (heat shock cognate 70 kDa protein 2), Eucgr.B01596 (probable xyloglucan endotransglucosylase/hydrolase protein 23), Eucgr.A01080 (glycine-rich RNA-binding protein), Eucgr.F00590 (snakin-2) and Eucgr.H03983 (major allergen Pru ar 1) (Additional file 2). A total of 1958 and 2127 down-regulated genes were specifically identified in *E. camaldulensis* and *E. grandis x urophylla*, respectively (lower panel of Fig. 3a).

Interestingly, we identified 6, 17, 12, 83, 28, 10, 9, 122 and 98 DEGs related to the embryogenesis, ethylene, auxin, RP, ZFP, HSP, histone, cell wall and TF, respectively, only in the dedifferentiation process of *E. camaldulensis* (Table 2, Additional file 2). The 6 embryogenesis related genes include 5 up-regulated genes (2 SE receptor kinase, 3 LEA) and 1 down-regulated gene encoding embryogenesis-associated protein EMB8. Among the 17 DEGs encoding auxin related products (Fig. 3b) specifically identified in *E. camaldulensis*, Eucgr.K02992 (auxin transporter-like protein 4) and Eucgr.C02984 (auxin-responsive protein IAA26) were down-regulated and Eucgr.H03171 (auxin-induced protein 22D) was up-regulated in *E. camaldulensis* but down-regulated in *E. grandis x urophylla* (Additional file 2). All the DEGs related to ethylene and specifically identified in the callus of *E. camaldulensis* were found to encode ER TFs, and 7 had reverse regulation in *E. camaldulensis* and *E. grandis x urophylla* (Fig. 3c). Interestingly, heat maps (Fig. 3d and e) showed that most of the *E. camaldulensis* specific DEGs encoding HSP and RP were down-regulated in the dedifferentiated tissue compared to the differentiated tissue. Although some of the DEGs encoding histone were more or less changed in *E. grandis x urophylla*, the difference of their expression was not significant as that in *E. camaldulensis* (Fig. 3f). Importantly, we found another 4 TF subfamilies including ASIL2, bHLH, MYB and WRKY were dysregulated only in *E. camaldulensis* (Fig. 3g). Further, the expression of MYB and WRKY TF genes were elevated during the dedifferentiation process of *E. camaldulensis*. The cell wall related DEGs specific to *E. camaldulensis* were involved in multiple process, such as “GO:0005199~structural constituent of cell wall”, “GO:0009664~plant-type cell wall organization” and “GO:0009832~plant-type cell wall biogenesis” (Additional file 3). It is interesting that Eucgr.F01000 (formin-like protein 5, log2FC = 1.65, *p* = 5.67E-05) were the only DEG involved in the “GO:0005199 ~ structural constituent of cell wall”. In addition, some other gene families were identified to be specifically differentially expressed in the SE of *E. camaldulensis* (Table 2, Additional file 3), such as 3 ABA related genes, 3 arabinogalactan protein genes, 4 ABC transporter genes and 21 abscisic stress-ripening protein genes.

### qRT-PCR validation

**Fig. 4:**
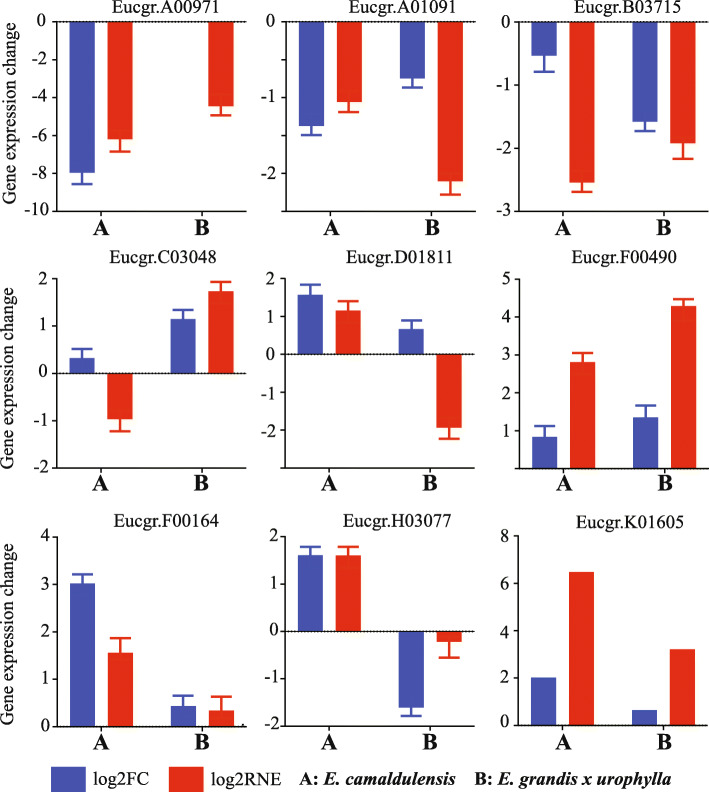
qRT-PCR validation. Log2FC and log2RNE represent the gene expression changes identified by the transcriptome sequencing and qRT-PCR, respectively

We used qRT-PCR to confirm the expression patterns of DEGs in the dedifferentiation process of *E. camaldulensis* and *E. grandis x urophylla*. We randomly selected 9 genes (Eucgr.A00971, Eucgr.A01091, Eucgr.B03715, Eucgr.C03048, Eucgr.D01811, Eucgr.F00490, Eucgr.F01164, Eucgr.H03077 and Eucgr.K01605) for the qRT-PCR experiment and the H2B gene was used as the internal control. The primer sequences for all these genes can be accessed in the Additional file 4. Each gene was replicated three time in every sample, so we performed 9 reactions in total for the differentiated and dedifferentiated tissues. Log2 fold change (log2FC) and log2 relative normalized expression (log2RNE) were used to present the gene changes detected by transcriptome sequencing and qRT-PCR, respectively. Overall, 14 (77.8%) out of 18 events were agreed by both qRT-PCR and deep sequencing (Fig. 4). Interestingly, the up-regulation of SE marker gene SERK1 was detected by qRT-PCR in both Eucalyptus species while transcriptome sequencing only detected its up-regulation in *E. camaldulensis*. However, other DEGs encoding SERK1 were identified with up-regulation in *E. grandis x urophylla* (Additional file 2). It is notable that the increase of WRKY TF (Eucgr.D01811) and the decrease of RP gene (Eucgr.A00971) in *E. camaldulensis* were confirmed by qRT-PCR. The dysregulation of Eucgr.B03715, Eucgr.C03048 and Eucgr.F00490 in *E. grandis x urophylla* were also confirmed. High agreement of gene expression patterns in transcriptome sequencing and qRT-PCR indicate that the genes identified in this study might be associated with the regenerative ability and the SE of Eucalyptus, which requires future experiments to be explored.

### SNPs and indels

We next identified gene variants (e.g., SNPs and small indels) in *E. camaldulensis* and *E. grandis x urophylla* using the transcriptome sequencing data. Initially, we obtained 97,504 and 75,582 variants in the differentiated and dedifferentiated tissues of *E. camaldulensis*, respectively (Table 3). After the variants supported by < 100 reads were filtered, we identified 97,974 variants for *E. camaldulensis*. Likewise, 72,208 and 66,311 variants were found in the differentiated and dedifferentiated tissues of *E. grandis x urophylla*, respectively, and they produced 78,977 variants after filtering variants with low supportive reads (Table 3). Comparison showed 49,527 variants shared by *E. camaldulensis* and *E. grandis x urophylla*, and 48,447 variants were specifically identified in *E. camaldulensis*. Then, we annotated the *E. camaldulensis* specific variants using ANNOVAR and found that 13,434 variants were functional, such as nonsynonymous, frameshift insertion, frameshift deletion, stop gain and stop loss variants (Table 3, Additional file 5). Interestingly, these 13,434 variants were derived from 4723 Eucalyptus genes, such as annexin (Eucgr.F02423, Eucgr.H00564), ARF guanine-nucleotide exchange factor GNOM (Eucgr.B03196), AP2-like ER TF (Eucgr.I00278, Eucgr.J02113), auxin response factors (Eucgr.G00076, Eucgr.C02178, Eucgr.C03293, Eucgr.J00923, Eucgr.F02090, Eucgr.D00264, Eucgr.E00888) and wall-associated receptor kinas-like (Eucgr.I01022). KEGG pathway analysis showed that 85 pathways including “longevity regulating pathway” (ko04211) and “Plant hormone signal transduction” (ko04075) were enriched by the of the *E. camaldulensis* specific mutated genes (Additional file 6).

**Table 3:** Gene variants identified in *E. camaldulensis* and *E. grandis x urophylla*

	*E. camaldulensis*		*E. grandis x urophylla*	
	A1	A2	B1	B2
Variants	97,504	75,582	72,208	66,311
DP^a^ > 100, *n* > 3	97,974		78,977	
Common	49,527			
Specific	48,447		29,450	
Functional variants	13,434		7684	
Involved genes	4723		3609	

^a^*DP* read depth

## Discussions

SE has become an efficient way for the propagation and SERK genes have been reported to play a key role during the SE of many plants, such as Arabidopsis [23], cacao [24], rice [25], sunflower [26], maize [27], grape [28] and pineapple [29]. In Arabidopsis, SERK1 was highly expressed during the formation of embryogenic cells and can be detectable in all cells of the developing embryo during early SE up to the formation of the heart stage [23]. SERK2 is significantly increased in the embryogenic callus and the maturation stage compared to non-embryogenic callus [30]. In the present study we identified the up-regulation of some SERK genes in the dedifferentiated tissue compared to differentiated tissue in both *E. camaldulensis* and *E. grandis x urophylla* (Additional file 2). Further, we found that 2 SERK gene members were specifically up-regulated in the dedifferentiation of *E. camaldulensis*. These results suggested that different members of the SERK gene family may have diverse functions. Singh and Khurana also observed the different expression patterns of SERK genes in wheat and reported that SERK genes were differentially expressed in response to different plant growth regulators [31]. For example, SERK2 and SERK3 can elicit the auxin-specific responses while SERK1 and SERK5 may be mediated by the signaling pathway of brassinosteroids. The functions of SERK genes in the SE of Eucalyptus require further experiments to be explored.

Méndez-Hernández reviewed the interactions between different plant growth regulators, mainly auxins, cytokinin, ethylene and abscisic acid (ABA), during the induction of SE [32]. Although how the cells initiate embryo formation is not clear, an irregular distribution of auxins must be established to initiate embryo formation. In the present study, three genes (Eucgr.B00948, Eucgr.G02187, Eucgr.A02229) encoding auxin efflux carrier were up-regulated only in the dedifferentiation process of *E. camaldulensis* (Table 2, Additional file 3). In addition, three genes encoding auxin response factors were specifically up-regulated in *E. camaldulensis*. We also identified DEGs related to other plant hormones, like late embryogenesis abundant protein (LEA) and abscisic acid (ABA). For example, twelve ABA related genes were dysregulated, including seven ABA receptor and five 8′-hydroxylase genes (Additional file 3). Among them, one ABA receptor and two ABA 8′-hydroxylase genes were specifically down-regulated in the dedifferentiated tissue of *E. camaldulensis*. Chen et al*,* reported that the ABA transcripts peaked at 8 h after ABA treatment and then significantly decreased at latter time points [33]. Although we lack multiple time points of the callus development, it can be speculated that the down-regulation of ABA genes might be necessary for the callus development of Eucalyptus. Interestingly, we found seven genes encoding LEA in the callus compared to the stem (Additional file 3). However, the expression level of down-regulated LEA gene (Eucgr.E00787) was much higher than the five up-regulated LEA genes. Among them, three (Eucgr.K01312, Eucgr.I01292, Eucgr.A02687) were specifically up-regulated in the callus of *E. camaldulensis*. The LEA genes play a pivotal role during the plant somatic embryogenesis process [34] and have been studied in some plant species like cotton [35], white spruce [36] and sweet orange [37].

In Eucalyptus, Marques identified QTLs for adventitious rooting, one QTL for sprouting ability and four QTLs for the stability of adventitious rooting [7]. Their results indicated that the phenotypic variation in these traits had a meaningful genetic component that relate to the capacity of vegetative propagation. We identified 13,434 variants in 4723 Eucalyptus genes specific to *E. camaldulensis* (Additional file 6), including two genes encoding SERK1 and SERK2, 25 auxin related genes (e.g., auxin transporter like protein, auxin-responsive protein, auxin efflux carrier component, auxin responsive factor) and 8 genes encoding ABA receptors (e.g., PYR1, PYL2, PYL4, PYL8, PYL9). Also, we found the *E. camaldulensis* specific mutated gene Eucgr.B03196 (the ARF guanine-nucleotide exchange factor GNOM, EMB30 or GN) has the potential to mediate the endosomal recycling, auxin transport and auxin-dependent plant growth [38]. Mutant EMB30 has the capacity of altering the cell wall in Arabidopsis [39]. It is also involved in the specification of apical-basal pattern formation in the early embryo and the root formation [40, 41]. Although our study lacks fundamental experiments, the pathways involved by the mutated genes in *E. camaldulensis* indicate that they might be associated with the SE and the regenerative ability of Eucalyptus.

TFs have been reported to function in cell division, cell growth, cell death, cell migration and organization during embryonic development, and respond to biotic and abiotic stresses. They can be another big category that affect the gene expression during the SE induction [32]. Some TFs have been reported to associate with the SE, including ABI3, WOX9–1, LEC, WUSCHEL, TALE, BBR-BPC and AP2 [32, 42]. In this study we identified some dysregulated TF genes exclusively in the callus of *E. camaldulensis* (Table 2, Additional file 2). Among them, ER, bHLH, MYB and WRKY TFs were up-regulated. QTL analysis has identified a WRKY TF gene related to the adventitious rooting from apple hardwood cuttings [43]. In Eucalyptus, WRKY33 was up-regulated in response to the fungal affection of *Chrysoporthe austroafricana* [44] and *Calonectria pseudoreteaudii* [45]. Also, WRKY TF was up-regulated in the *Eucalyptus camaldulensis* seeding subjected to the water stress [46].

Jozef Šamaj discussed the structural, physiological and functional aspects connected to the role of the cell wall during embryogenesis in plant [47], including the cell wall components arabinogalactan proteins and pectins. We identified 274 and 251 DEGs related to cell wall in *E. camaldulensis* and *E. grandis x urophylla*, respectively (Table 2, additional file 3). Among them, 5 and 10 genes have the capacity of encoding fasciclin-like arabinogalactan proteins and pectinesterase, respectively (additional file 3). Interestingly, Eucgr.A01158 (fasciclin-like arabinogalactan protein 11), a protein involved in the plant-type secondary cell wall biogenesis, was found with up-regulation in the SE of *E. camaldulensis* but down-regulation in the SE of *E. grandis x urophylla* (additional file 3). Compared to *E. camaldulensis*, more up-regulated genes encoding pectinesterase were found in the SE of *E. grandis x urophylla* (additional file 3). The functions of cell wall related genes need future experiments to be explored.

Furthermore, RP [48], HSP [49], and histone [50, 51] have been reported to function in the vegetative propagation in plants. While very few studies have been demonstrated to investigate their functions and associations in the SE of Eucalyptus, our results indicate that the dysregulation of these genes and pathways like metabolisms involved by these genes might play important roles in the SE of Eucalyptus.

## Conclusions

In conclusion, we studied the transcriptome profiles during the SE of two Eucalyptus species – *E. camaldulensis* and *E. grandis x urophylla*. Our results showed dysregulated genes from some gene families like auxin, ethylene, SERK, RP, ZFP, HSP, histone, ABA, LEA and TF might play key roles in the SE and the regenerative ability of Eucalyptus. Also, we called SNPs and small indels in *E. camaldulensis* and *E. grandis x urophylla*, and it was revealed that genetic variants may also contribute to the SE and the regenerative ability of Eucalyptus. This is the first time to study the SE and the dedifferentiation in Eucalyptus using transcriptome sequencing. It will improve our understanding of the molecular mechanisms during the SE in Eucalyptus. Our findings provide a valuable resource for future studies in the field of Eucalyptus and, more importantly, will benefit the Eucalyptus breeding program.

## Methods

### Plants

The original seeds of *E. camaldulensis* (high regenerative ability, voucher ID: c0009) and *E. grandis x urophylla* (low regenerative ability, voucher ID: j0017) were obtained from the wild in 1984 and no permissions were required to collect these plants. Then, they were confirmed by a senior botanist Prof. Dongyun Xiang and were maintained in the experimental fields of Guangxi Forestry Research Institute. The second generation of in vitro tissue-culture induced seedlings of *E. camaldulensis* and *E. grandis x urophylla* were maintained on the MS medium supplemented with 20 mg/L Ca (NO_3_)_2_, 0.5 mg/L 6-BA and 0.1 mg/L IAA until 2 to 3 cm height. The second to the third stems from the stem tip of the seedlings were obtained and cut into 0.3 ~ 0.5 cm segments. About 60 segments of each Eucalyptus species were then transferred onto the induction MS medium (supplemented with 20 mg/L Ca (NO_3_)_2_, 1 mg/L KT and 0.5 mg/L 2,4-D) and maintained in dark at 28 ± 2 °C for 10 days. Every day the callus was weighted and measured for the growth curve analysis. Stem (0 d) and complete callus (10 d) were used as the differentiated and dedifferentiated sample, respectively. The induction experiment was replicated three times.

### RNA isolation, library construction and transcriptome sequencing

Differentiated and dedifferentiated tissues (100 mg) were collected for the total RNA isolation using the TRIzol reagent (Invitrogen) according to the manufacturer’s protocol, as previously described [52, 53]. Then, the quantity and quality of the total RNA were determined by the Agilent 2100 Bioanalyzer and equal amount of total RNA (1 μg) was used to construct the libraries for BGISEQ-500 RNA-Seq. Briefly, magnetic oligo (dT) beads were used to enrich the ploy(A) mRNAs, which were then fragmented into ~ 200 bp. Then, the fragments were used for the double strand cDNA library construction by random hexamer (N6) primers, followed by the end repair using phosphate at the 5′ end and sticky ‘A’ at the 3′ end. Next, sequencing primers were ligated to build the final cDNA library, which was then sequenced on the BGISEQ-500 RS platform at BGI-Shenzhen with paired-end 150 strategy.

### Genome mapping and gene expression profiling

Raw data of each sample was processed by the trim_galore (https://www.bioinformatics.babraham.ac.uk/projects/trim_galore/) to remove sequencing adaptors, low quality reads and contamination reads. Then, the clean data was quality-controlled by fastqc (http://www.bioinformatics.babraham.ac.uk/projects/fastqc/) and then aligned to the *E. grandis* genome (v2.0, https://plantgenie.org) using Hisat2 with default parameters [54]. Next, Stringtie (v1.3.4d) was used to profile gene expression in each sample [54]. TPM (transcripts per million reads mapped) method was used to normalize the gene expression for each sample. The numbers of reads aligned to Eucalyptus genes were calculated by htseq-count, as described [55].

### Differential expression analysis

We used edgeR to identify differentially expressed genes in stem and callus of the two Eucalyptus species. Stringent cut-offs were employed to select the DEGs, including log2 fold change (log2FC) > 1 or < − 1, *p*-value < 0.05 and false discovery rate (FDR) < 0.1 [49].

### Variant calling

We used strelka (2.9.10–4-gd737744) to call the variants, including SNPs and small indels, in the stem and callus tissues of both *E. camaldulensis* and *E. grandis x urophylla* [56]. Then, variants passed the program filter and identified in all three replicates were kept for downstream analysis. We next filtered the variants by the read depth (> 100) and obtained the final variants for *E. camaldulensis* and *E. grandis x urophylla* samples. The variants were annotated by ANNOVAR, as previously described [55], and variants from noncoding regions and synonymous variants were discarded.

### Functional analysis

To analyze the potential pathways and biological processes involved by the DEGs and mutated genes, we first annotated the *E. grandis* genes using the KEGG pathway and Gene Ontology databases, as previously described [53]. Then, enriched KEGG pathways and GO terms by the DEGs were identified using two statistical values: p-value (calculated by Fisher’s exact test, < 0.05) and q-value (calculated by the R package ‘qvalue’, < 0.05), as previously described [53].

### qRT-PCR

We performed the quantitative real-time PCR to confirm the expression changes of candidate genes. A total of nine genes (Eucgr.A00971, Eucgr.A01091, Eucgr.B03715, Eucgr.C03048, Eucgr.D01811, Eucgr.F00490, Eucgr.F01164, Eucgr.H03077 and Eucgr.K01605) were randomly selected and the H2B gene was used as the internal control. Primers were predicted using the Primer3 and synthesized at BGI-Shenzhen. The procedure of qRT-PCR was same as a previous study [57]. We performed three times for each gene in every replicate (*n* = 9). After the Ct values were calculated, we used ΔCt value to present the gene expression. Then, ΔΔCt was used to show the difference of a gene in the callus compared to the stem. Relative normalized expression (RNE) was used to show the gene expression change: *RNE* = 2^−ΔΔCt^.

## Supplementary Information

**Additional file 1. **Gene expression profiles of differentiated and dedifferentiated tissues of *E. camaldulensis* and *E. grandis x urophylla*.

**Additional file 2. **Differentially expressed genes in the dedifferentiated tissues compared to the differentiated tissues of *E. camaldulensis* and *E. grandis x urophylla*.

**Additional file 3. **Cell wall related DEGs identified in the SE of *E. camaldulensis* and *E. grandis x urophylla*.

**Additional file 4.** Primers used for qRT-PCR.

**Additional file 5. **Gene variants specifically identified in *E. camaldulensis*.

**Additional file 6. **KEGG pathway enriched by the mutated genes specifically identified in *E. camaldulensis*.

## Data Availability

The raw sequencing data can be accessed from the NCBI Sequence Read Archive (SRA) platform (http://trace.ncbi.nlm.nih.gov/Traces/sra/) under the accession number PRJNA634476.

## Abbreviations

QTL: Quantitative trait loci
SERK: Somatic embryogenesis receptor kinase
HSP: Heat shock protein
TF: Transcription factor
LEA: Late embryogenesis abundant protein
TPM: Transcripts per million reads
DEG: Differentially expressed gene
GO: Gene ontology
KEGG: Kyoto Encyclopedia of Genes and Genomes
log2FC: log2 fold change
log2RNE: log2 relative normalized expression
SNP: Single nucleotide polymorphism
